# Expression of the H_2_O_2_ Biosensor roGFP-Tpx1.C160S in Fission and Budding Yeasts and Jurkat Cells to Compare Intracellular H_2_O_2_ Levels, Transmembrane Gradients, and Response to Metals

**DOI:** 10.3390/antiox12030706

**Published:** 2023-03-13

**Authors:** Laura de Cubas, Jorge Mallor, Víctor Herrera-Fernández, José Ayté, Rubén Vicente, Elena Hidalgo

**Affiliations:** 1 Oxidative Stress and Cell Cycle Group, Universitat Pompeu Fabra, C/ Doctor Aiguader 88, 08003 Barcelona, Spain; 2 Laboratory of Molecular Physiology, Universitat Pompeu Fabra, C/ Doctor Aiguader 88, 08003 Barcelona, Spain

**Keywords:** H_2_O_2_ sensor, roGFP-Tpx1.C169S, Jurkat, fission yeast, H_2_O_2_ concentrations, zinc

## Abstract

Intracellular hydrogen peroxide (H_2_O_2_) levels can oscillate from low, physiological concentrations, to intermediate, signaling ones, and can participate in toxic reactions when overcoming certain thresholds. Fluorescent protein-based reporters to measure intracellular H_2_O_2_ have been developed in recent decades. In particular, the redox-sensitive green fluorescent protein (roGFP)-based proteins fused to peroxiredoxins are among the most sensitive H_2_O_2_ biosensors. Using fission yeast as a model system, we recently demonstrated that the gradient of extracellular-to-intracellular peroxides through the plasma membrane is around 300:1, and that the concentration of physiological H_2_O_2_ is in the low nanomolar range. Here, we have expressed the very sensitive probe roGFP2-Tpx1.C169S in two other model systems, budding yeast and human Jurkat cells. As in fission yeast, the biosensor is ~40–50% oxidized in these cell types, suggesting similar peroxide steady-state levels. Furthermore, probe oxidation upon the addition of extracellular peroxides is also quantitatively similar, suggesting comparable plasma membrane H_2_O_2_ gradients. Finally, as a proof of concept, we have applied different concentrations of zinc to all three model systems and have detected probe oxidation, demonstrating that an excess of this metal can cause fluctuations of peroxides, which are moderate in yeasts and severe in mammalian cells. We conclude that the principles governing H_2_O_2_ fluxes are very similar in different model organisms.

## 1. Introduction

Hydrogen peroxide (H_2_O_2_) participates in signaling events, but it is also linked to cell toxicity. The generation and scavenging of H_2_O_2_ can lead to different concentrations of H_2_O_2_ in space and time. These fluctuating levels of peroxides regulate signaling cascades, which indeed control physiological processes as well as antioxidant pathways (for reviews, see [1,2,3,4]).

As mentioned above, the intracellular levels of H_2_O_2_ depend on synthesis but also on scavenging activities. Biological membranes have limited permeability to H_2_O_2_, and compartment-specific scavenging activities create gradients across membranes. Not only that, but the effect of extracellularly added compounds such as metals has been proposed to alter the intracellular H_2_O_2_ levels. That is the case of zinc.

It is likely that about 5–10% of eukaryotic proteomes are zinc-containing proteins, distributed in the cytosol and all major organelles [5]. Many of these proteins are highly abundant, and therefore cells need an important supply of zinc to yield a functional ‘zinc-proteome’. As a reference, the amount of zinc used for optimal growth of fission yeast in minimal medium is 1.4 µM zinc sulfate [6]. This metal is not redox-active, but it is essential due to its contribution to the structure and/or function of many proteins and its participation as a second messenger in signaling pathways. Zinc has to be internalized by cells from the environment using zinc-specific transporters [7,8]. It can be toxic when present in excess, probably through uncontrolled binding to intracellular targets and/or competition with other metals at their active sites. To avoid toxicity, cytosolic zinc content is tightly regulated by zinc-binding proteins and transporters that mobilize zinc from the cytosol to cellular compartments such as the lysosome (in animal cells) or vacuole (in yeasts) [9,10], or the endoplasmic reticulum [11,12]. At least in both budding and fission yeasts, zinc accumulation in these intracellular compartments is the main system both for sequestering available zinc (for detoxification and storage purposes) and liberating it during deprivation situations. Organisms have developed homeostatic transcriptional control pathways to coordinate the internalization and buffering of zinc by regulating the expression of zinc-binding proteins and zinc transporters depending on metal levels [7,13,14,15].

Again in both yeasts, zinc is only toxic at extremely high concentrations of the metal, in the millimolar range [9,16], while 100 µM is sufficient to trigger toxicity to Jurkat and other human cell lines [8]. The lack of control systems to activate or repress the transporters described above can lead to sensitivity or further resistance to those extreme concentrations. In some cases, toxicity has been associated with the production of reactive oxygen species [9,17,18,19,20,21].

Due to all of the above, measuring H_2_O_2_ fluctuations in living cells in different sub-cellular locations has been the goal of many laboratories for decades. Many small permeable fluorescent dyes are used to measure intracellular peroxides, with the boronate ester/boronic acid group-based probes among them [22,23]. Alternatively, two popular families of genetically encoded redox reporters are the redox-sensitive green fluorescent protein (roGFP)-based proteins [24], in which the roGFP moiety is fused to peroxidases [25,26,27] and the HyPer derivatives [28,29].

The roGFP moiety has several substitutions on surface-exposed domains, so that disulfide bonds can be formed in response to oxidants and change the fluorescence properties. In particular, the dual-excitation, single-emission properties of this fluorophore can be used for ratiometric measurements, dismissing the effects caused by the intracellular concentration of the biosensor. The specificity and sensitivity of roGFP to sense H_2_O_2_ were improved by fusing a peroxiredoxin, Tpx1, lacking its resolving cysteine [27]. This mutation enhances sensitivity since it eliminates competition by the thioredoxin-based reducing system and improves oxidation transfer from Tpx1 to roGFP.

Using the fission yeast *Schizosaccharomyces pombe* as a model system, we recently estimated that the gradient of extracellular-to-intracellular peroxides is on average 300:1 (a permeability gradient of 40:1 is enhanced by intracellular scavenging up to 300:1; reaching this gradient takes longer at high concentrations of applied peroxides) [30]. Taking advantage of this knowledge, we then established that roGFP2-Tpx1.C169S is able to detect intracellular H_2_O_2_ fluctuations in the low nanomolar (~3–6 nM) range. Since our probe is based on the main H_2_O_2_ *S. pombe* scavenger Tpx1 [31], we proposed that the capacity of this reporter to sense peroxides coincides with the steady-state levels of peroxides in the cytosol of wild-type cells, which are probably below and close to 3–6 nM. The basal concentration of H_2_O_2_ as well as the gradients formed between external and cytosolic H_2_O_2_ when exposing these cells to H_2_O_2_ in the medium was characterized in human K−562 cells using another H_2_O_2_ reporter, HyPer, as a probe, and applying mathematical equations, yielding very similar results to the ones proposed for fission yeast [32].

To probe or dismiss whether these two properties, the 300-to-1 gradients across the plasma membrane and the steady-state peroxide levels under basal conditions, are universal, we have expressed roGFP2-Tpx1.C169S in three model systems: fission and budding yeast as well as human Jurkat cells. The high concentration of the reporters in all model systems allowed us to monitor probe oxidation directly in diluted cell suspensions, mimicking physiological situations. We used a fluorescence plate reader to monitor physiological probe oxidation since both the unicellular yeasts systems and Jurkat cells grow in suspension. In all three systems, the steady-state levels of probe oxidation were very similar, around 40–50%, suggesting that the intracellular cytosolic peroxide levels are comparable. Probe oxidation was also monitored upon the addition of extracellular H_2_O_2_ with similar results in the three systems, which indicates that the peroxide gradients across the plasma membrane are also comparable. Finally, we tested whether zinc levels, often associated with the generation of reactive oxygen species, cause oxidation of the probe. Indeed, we detected a small but significant oxidation of roGFP2-Tpx1.C169S in both yeast systems, while the probe suffered continuous and dose-dependent oxidation, suggesting toxicity in Jurkat cells. At least in fission yeast, this zinc-dependent intracellular burst of H_2_O_2_ is caused by the over-activation of Sod1, the Cu/Zn superoxide dismutase. We conclude that the principles governing H_2_O_2_ homeostasis are maintained in three different eukaryotic model systems.

## 2. Materials and Methods

### 2.1. Generation of Plasmids Used in This Study

Plasmid p407.C169S, allowing the expression of roGFP2-Tpx1.C169S, has been previously described [27]. For plasmids expressing the biosensor in *Saccharomyces cerevisiae* and Jurkat cells, roGFP2-Tpx1.C169S was PCR-amplified from p407.C169S and cloned into pNOPGFP2L (pRS425-NOP1p::GFP(S65T) (K. Hellmuth and E. Hurt, unpublished results) or MSCV puromycine [33], yielding plasmids p791 and p797, respectively.

### 2.2. Fission and Budding Yeasts Growth Media and Genetic Manipulations

For all the experiments, *S. pombe* cells 972 (*h*^−^; [34]), or the leucine auxotrophic strains HM123 (*h*− *leu1–32*, lab stock), SG5 (*h*+ *tpx1::natMX6 leu1–32*; [35]), and JM18 transformed with plasmid p407.C169S were grown in filtered minimal medium (MM) at 30 °C as described previously [27]. Strain JM18 (*h*^−^ *sod1::kanMX6 leu1–32*) was constructed by homologous recombination using linear DNA obtained by PCR-amplification with primers flanking the *sod1* open reading frame and pFA6a-kanMX6 [36] as a template. *S. cerevisiae* strain BY4741 (*MATa*, *his3*Δ*1 leu2*Δ*0 met15*Δ*0 ura3*Δ*0*, Invitrogen, Waltham, MA, USA) was grown in synthetic defined (SD) media (Formedium, Swaffham, UK) supplemented with synthetic complete mixture drop-out plus uracil, leucine and histidine; BY4741 transformed with p791 was only supplemented with uracil and histidine. 

### 2.3. Growth Media for Jurkat Cells, and Selection of a Stable Cell Line Expressing roGFP2-Tpx1.C169S

Cells were maintained in RPMI 1640 medium (Gibco, Waltham, MA, USA) supplemented with 10% FBS and 1% penicillin/streptomycin, and 0.75 µg/mL puromycin when specified. Cells were grown at 37 °C in a humidified 5% CO_2_ atmosphere. For creating the stable line expressing roGFP2-Tpx1.C169S, 0.5 × 10^6^ cells were seeded into 6-well plates and transfected following Lipofectamine 3000 (ThermoFisher, Waltham, MA, USA) following manufacturer instructions. After two days we added puromycin as indicated before, and two days later we selected GFP-positive cells by cell-sorting. Cells were centrifuged at 1200 rpm for 5 min and resuspended in 500 µL of fresh media prior to sorter selection. They were sorted in a FacsAria (BD Biosciences, Franklin Lakes, NY, USA) system. Cells were gated from a forward versus side scatter plot. A viability dye (DAPI or TO-PRO-3) was also used; negative staining of either of these dyes was used to exclude death. Then, the green-fluorescent-positive cells, compared to the wild type not transfected, were selected and recovered in 400 µL of filtered media from exponentially growing Jurkat cells in a 96-well plate. Cells were progressively expanded by adding fresh medium until reaching a final initial volume of 2 mL in 6-well plates. Then, the puromycin treatment plus cell sorting process was repeated once again until we reached a single population expressing green fluorescence (roGFP2-Tpx1.C169S). The stable line expressing roGFP2-Tpx1.C169S was cultured in a whole medium supplemented with puromycin to maintain the selection.

### 2.4. Growth of Strains Expressing roGFP2-Tpx1.C169S for Fluorescence Determination

For *S. pombe*, standard MM-based early stationary phase pre-cultures were diluted in filtered MM to reach an OD_600_ of 1 after 4–5 duplications. The fluorescence of 190 µL of these cultures, at an OD_600_ of 1, was directly monitored in 96-well plates in a FLUOstar OMEGA (BMG Labtech, Otenberg, Germany) as described below. When specified, cells were centrifuged for 1 min at 1500 rpm and resuspended in pre-warmed filtered MM previous to fluorimeter measurements. 

For *S. cerevisiae*, cells were grown in SD media till an OD_600_ of 1. Cultures were centrifuged and resuspended in the same volume of pre-warmed *S. pombe* media (filtered MM, as above). Amounts of 190 µL of these cell suspensions were plated in 96-well plates.

For Jurkat cells, cultures were grown until confluence, then centrifuged at 1200 rpm for 5 min, and resuspended in pre-warmed isotonic (ISO) media containing 140 mM NaCl, 2.5 mM KCl, 1.2 mM CaCl_2_, 0.5 mM MgCl_2_, 5 mM glucose, and 10 mM Hepes (adjusted to pH 7.3 with Trizma base and osmolarity to 300–310 mOsm with D-Mannitol) [37]. Based on the volume of Jurkat cells, cell suspensions with an OD_600_ of 0.5 corresponded to ~3 × 10^6^ cells/mL. Amounts of 190 µL of these suspensions were plated for fluorescence measuring in 96-well plates.

### 2.5. Live-Cell Measurements of Basal and Induced Oxidation of roGFP2-Tpx1.C169S 

roGFP2-Tpx1.C169S exhibits two excitation maxima at 400 nm and 475–490 nm. The fluorescence emission is monitored at 510 nm. We used excitation filters of 400–10 and 485BP12, combined with emission filter EM520. The experiments were performed as previously described [27]. Briefly, 190 µL of cultures was transferred as described above to a 96-well imaging plate (Krystal Microplate™ 215003, Porvair Sciences, Norfolk, UK) in as many wells as treatments to be tested. We measured the excitation wavelength during 4 cycles after which we applied 10 µL of the corresponding treatments to accomplish the final concentrations indicated in the figures. Most yeast experiments were performed at 30 °C and 700 rpm shaking for 15 s after each cycle, except when compared with Jurkat cell suspensions. In that case, the recording was at 37 °C and 100 rpm shaking for 5 s. Each strain or cell type was treated in two wells with 1 mM H_2_O_2_ and 50 mM dithiothreitol (DTT) as controls of fully oxidized or fully reduced reporters, afterward applying the formula that calculates de OxD (see below).

For *S. pombe* (Figures 3e and 4), the fluorescence of a wild-type strain, 972, not expressing the biosensor and treated with H_2_O (for subtractions from all the treatments including H_2_O_2_ or zinc) or DTT (only for subtracting from the DTT-treated samples) was subtracted from the fluorescence values from cells expressing the reporter, to minimize the effects of the endogenous green fluorescence on calculations (notably, H_2_O_2_ or zinc treatments did not alter the intrinsic fluorescence of empty 972, but DTT did). In the case of *S. cerevisiae* and Jurkat cells, tests were performed to confirm that these treatments were not affecting fluorescence levels over empty strains; for the final calculations, no subtraction was performed to decrease the number of strains and to have the two or three model systems expressing the reporter coming from several biological replicates monitored within the same 96-well plate. We determined the degree of sensor oxidation (OxD) as described in the following Equation (1):

(1) $$\mathrm{OxD}=\frac{\left(I_{\mathrm{sample}\;488}\times I_{\mathrm{DTT}405})-{(I}_{\mathrm{sample}\;405}\times I_{\mathrm{DTT}\;488}\right)}{{(I}_{\mathrm{sample}\;405}\times I_{H_{2}O_{2}\;488})-{(I}_{\mathrm{sample}\;405}\times I_{\mathrm{DTT}\;488})-(I_{\mathrm{sample}\;488}\times I_{H_{2}O_{2}\;405})+(I_{\mathrm{sample}\;488}\times I_{\mathrm{DTT}\;405})}$$

where ‘I’ represents the fluorescence intensity at 510 nm after excitation at either 405 nm or 488 nm of the sample treated with H_2_O_2_ or ZnSO_4_ at a given time and concentration. The control intensity values for maximum and minimum determination, H_2_O_2_, and DTT, respectively, were selected 10 min after treatment addition. For graphs depicting the behavior of 405 and 488 wavelengths before and after different treatments, raw data provided by the fluorimeter was used. For Figure 3b, the data were corrected by subtracting the intensity of Jurkat expressing the reporter treated with H_2_O as control. 

### 2.6. Western Blot of roGFP2-Tpx1.C169S

Yeast strains were grown until an OD_600_ of 0.5, and protein extracts were prepared by TCA precipitation as previously described [38]. For Jurkat, cell suspensions containing 5 × 10^6^ cells were centrifuged and resuspended in 50 µL of the same buffer as yeasts, containing 1% SDS, 100 mM Tris·HCl (pH 8), and 1 mM EDTA. Lysates were boiled under reducing conditions and loaded in 10% SDS-polyacrylamide gel. Immunoblots were performed as previously described [39], using an anti-GFP monoclonal antibody (Takara, Kusatsu, Japan), followed by a secondary anti-mouse Starbright blue 700 fluorescent antibody (BioRad, Hercules, CA, USA). For both antibodies, skim milk was used as a membrane-blocking agent. Ponceau was used as a loading control [39].

### 2.7. Microscopy

Cells grown as previously described were collected by centrifugation and resuspended in MM (yeasts) or ISO (Jurkat), loaded onto slides, and analyzed by fluorescence microscopy. We used a Nikon Eclipse 90i microscope equipped with differential interference contrast optics, a PLAN APO VC 100x 1.4 oil immersion objective, an ORCA-II-ERG camera (Hamamatsu, Japan), the image acquisition software Metamorph 7.8.13 (Gataca Systems, Massy, France), and an LED illumination Cool LED pE-300lite. All images were acquired with a green fluorescence filter (ex. 460–500 em. 510–56). Analysis was performed using Fiji software.

### 2.8. Statistics

**Figure 1:**
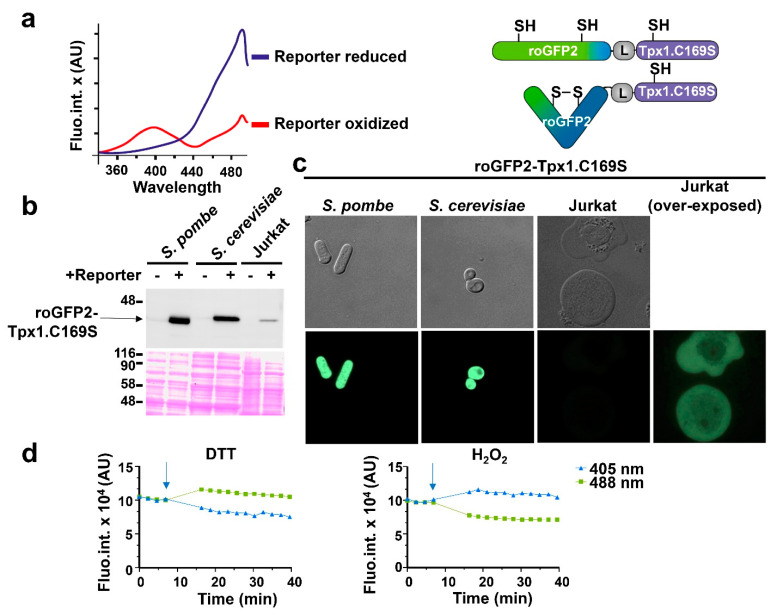
Expression of the H_2_O_2_ biosensor roGFP2-Tpx1.C169S in fission and budding yeasts and in Jurkat T cells. (**a**) Left: representation of the fluorescence excitation spectra of reduced and oxidized roGFP2-Tpx1.C169S. The blue line is the fully reduced reporter form and the red line represents the fully oxidized form. X-axis: excitation wavelengths. Y-axis: AU (arbitrary units) of emission fluorescence at 520 nm. Right: representation of the reduced (up) and the oxidized (down) states of roGFP2-Tpx1.C169S. Upon H_2_O_2_ treatment, cysteine 48 of Tpx1 oxidizes to sulfenic acid and afterward oxidizes roGFP2, which forms a disulfide bond. (**b**) Western blot of the three used models. Cells expressing or not the probe roGFP2-Tpx1.C169S were processed as described in Materials and Methods. Strains of *S. pombe* (HM123) and *S. cerevisiae* (BY4741) were transformed with plasmids p407.C169S and p791 (+) and processed in parallel with their counterparts not expressing the reporter (972 or BY4741, respectively) (−). For Jurkat T cells, extracts from cells stably transfected or not with plasmid p797 to express roGFP2-Tpx1.C169S were grown and processed as described in Materials and Methods. Ponceau staining was used as a loading control. The sizes of molecular weight markers (in kDa) are indicated. (**c**) Micrographs of the three models. Cells were grown in their corresponding media, concentrated, and resuspended in MM (yeasts) or ISO (Jurkat), and loaded on slides for image acquisition. Image brightness and contrast were not adjusted, except when indicated (Jurkat (over-exposed)). (**d**) Graphs showing the time-dependent (X-axis) emission fluorescence (Y-axis, fluorescence arbitrary units, AU) upon excitation at 405 or 488 nm of Jurkat T cells expressing the reporter roGFP2-Tpx1.C169S. The arrows indicate the time of addition of 1 mM of H_2_O_2_ or 50 mM of DTT.

For fluorescence probe oxidation quantification of cells in culture, OxD, one independent culture of the strain of interest was grown for each replicate. In all figure panels, values of the mean of n = 3 or n = 4 are represented. For the statistical analysis, a ratio paired *t*-test was performed. In supplementary figures, error bars for all the experiments (standard deviation, S.D.) are represented. Only when showing the effect of separated wavelengths on different treatments (Figures 1d and 3b,d,f) only one replicate was used, as fluorescence arbitrary units are depicted.

## 3. Results

### 3.1. Expression of the H_2_O_2_ Biosensor roGFP2-Tpx1.C169S in Budding and Fission Yeast and in Jurkat Cells 

As described in the Introduction, we recently designed an H_2_O_2_ biosensor, roGFP2-Tpx1.C169S, and expressed it in fission yeast cells [27] (Figure 1a). As with all the roGFP derivatives, it displays two excitation maxima, which change with H_2_O_2_-dependent cysteine oxidation, and one emission wavelength, and this allows a concentration-independent, ratiometric quantification of probe oxidation. We generated plasmids to express the reporter in budding yeast and Jurkat cells, under the control of the constitutive *nop1* and MCV promoters, respectively. The relative concentrations of the probe in the three model systems were determined by Western blot analysis (Figure 1b) and fluorescence microscopy (Figure 1c). The concentration of roGFP2-Tpx1.C169S was higher in fission yeast than in *S. cerevisiae* or Jurkat cells, using anti-GFP antibodies. As explained above, concentration should not affect the interpretation of the results.

The behavior and fluorescence properties of roGFP2-Tpx1.C169S expressed in the three cell types were very similar. Thus, the addition of the strong reductant, DTT, to Jurkat cell suspensions caused a decrease in fluorescence emission at the excitation wavelength of 405 nm, and an increase at 488 nm, while 1 mM H_2_O_2_ caused the inverse behavior in probe excitation properties (Figure 1d). The same was observed with fission and budding yeast cell cultures. 

### 3.2. The Response to Extracellular Peroxides and the Steady-State Levels of H_2_O_2_ Are Very Similar in the Three Eukaryotic Models

**Figure 2:**
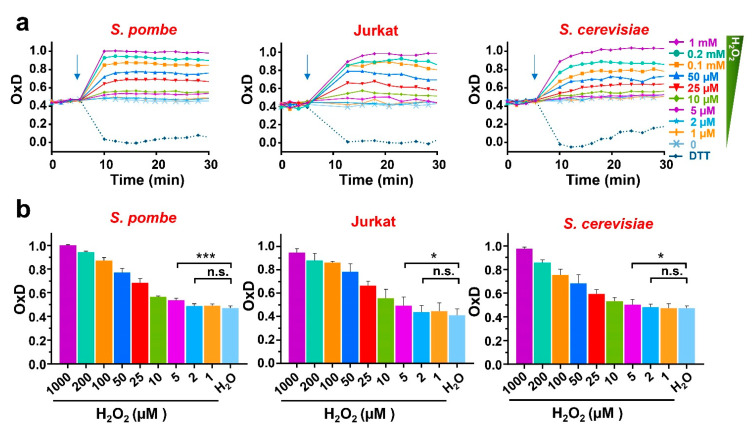
H_2_O_2_-dependent oxidation of roGFP2-Tpx1.C169S expressed in *S. pombe*, Jurkat T cells, and *S. cerevisiae*. Wild-type of *S. pombe* (HM123), *S. cerevisiae* (BY4741), or Jurkat T cells, transformed with p407.C169S, p791, or p797 to express roGFP2-Tpx1.C169S, were grown in their own media (MM-, SD-, or RPMI 1640-supplemented), centrifuged and resuspended in MM (both yeasts) or ISO (Jurkat) for a final OD_600_ of 1 (both yeasts) or 0.5 (Jurkat cells). Then, 190 µL of these cell suspensions were plated in 96-well imaging plates, and incubation and fluorescence recording were initiated at 37 °C with 100 rpm shaking. After 4 cycles, the indicated treatments of DTT or H_2_O_2_ were applied (indicated with arrows). The degree of probe oxidation, or OxD (amount of probe oxidation per 1), is indicated over time. (**b**) Bar graphs represent the average of 3 OxD values from the 12 to 17 min time points for each treatment and cell type represented in (**a**). Statistical significance was calculated between the indicated samples with a ratio paired Student’s *t*-test with *p*-values of 0.05 (*) and 0.001 (***); n.s., non-significant. For each strain, average data from four biological replicates are shown, with error bars (S.D.) of (**a**) displayed in Figure S1.

To test the response to extracellular peroxides in the three model systems, we applied different concentrations of H_2_O_2_ to cell suspensions in 96-well plates and quantitated the degree of probe oxidation in cells in culture. To use the same conditions and compare the results from the three model systems, we performed all the readings at 37 °C and with moderate shaking. As shown in Figure 2a,b and Figure S1, the oxidation of roGFP2-Tpx1.C169S expressed in *S. pombe*, *S. cerevisiae*, and Jurkat cells was maximal with 1 mM H_2_O_2_, and it reached ~60–65% oxidation upon 25 µM extracellular H_2_O_2_ (inverted red triangles in Figure 2a; red bar in Figure 2b). The reduction in the probes was also similar when longer time points were analyzed in the three systems. These results suggest that the concentrations of intracellular peroxides upon addition of H_2_O_2_ to the cell media are very similar in the three cell types, and therefore that the gradient of extracellular-to-intracellular peroxides is in the order of 300-to-1, as previously described in fission yeast. We proposed that the starting levels of oxidation of the roGFP2-Tpx1.C169S probe, OxD_0_, are an indicator of H_2_O_2_ steady-state levels in different fission yeast backgrounds, moving up from 50% in a wild-type background to 70–80% in a strain lacking the main H_2_O_2_ scavenger, Tpx1 [27]. As shown in Figure 2, the OxD_0_ of the roGFP2-Tpx1.C169S expressed in all three backgrounds were very similar at these experimental settings, around 45%, suggesting that the peroxide levels are similar, probably in the low nanomolar range. 

### 3.3. Addition of Extracellular Zinc Causes Intracellular Bursts of H_2_O_2_ in the Three Eukaryotic Models

**Figure 3:**
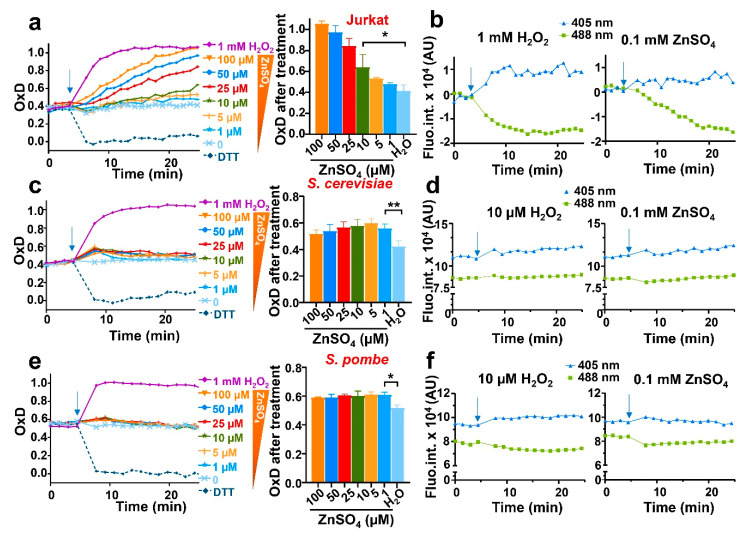
The addition of extracellular zinc causes intracellular bursts of H_2_O_2_ in the three eukaryotic models. Cell suspensions of Jurkat T cells (**a**,**b**), BY4741 (*S. cerevisiae*) (**c**,**d**), or HM123 (*S. pombe*) (**e**,**f**) expressing roGFP2-Tpx1.C169S were treated with 1 mM of H_2_O_2_ (as a control) or with the indicated concentrations of ZnSO_4_, ranging from 1 to 100 µM. Probe oxidation was determined as in Figure 2. (**a**) Jurkat experiments were performed as previously described at 37 °C with 100 rpm shaking. The middle panel (bar graph) represents the OxD at min 25. (**b**) Graphs showing the time-dependent (X-axis) emission fluorescence (Y-axis, fluorescence arbitrary units, AU) of the reporter in Jurkat upon excitation at 405 or 488 nm. (**c**–**f**) Yeast experiments were performed at 30 °C and 700 rpm shaking. The middle panel (bar graph) represents the OxD at the first time point after treatment. (**d**,**f**) Graphs showing the time-dependent (X-axis) emission fluorescence (Y-axis, fluorescence arbitrary units, AU) of the reporter in yeasts upon excitation at 405 or 488 nm. In all graphs, the time of addition is indicated with arrows. Statistical significance was calculated with a ratio paired Student’s *t*-test between pointed-out samples with *p*-values of 0.05 (*) and 0.01 (**). For each strain, average data from three biological replicates are shown, with error bars (S.D.) of left panels (**a**,**c**,**e**) displayed in Figure S2.

As a proof-of-concept of the usefulness of expressing our biosensor in different model systems, and with the aim of probing or dismissing H_2_O_2_ production as a secondary molecule explaining the toxicity of zinc reported in Jurkat cells, we treated our three model systems expressing roGFP2-Tpx1.C169S with increasing concentrations of zinc, ranging from low to high micromolar concentrations; again, the upper range of these concentrations triggered toxicity to Jurkat cells but not to fission or budding yeast. As shown in Figure 3a and Figure S2, increasing concentrations of the metal added to the media caused progressive oxidation of the probe expressed in Jurkat cells, indicative of intracellular H_2_O_2_ generation. To demonstrate that probe oxidation is caused by intracellular peroxides and not by non-specific binding of the metal to the biosensor, we monitored independently the emission at the two excitation maxima. Indeed, upon zinc addition we observed an increase in fluorescence at the excitation wavelength of 405 nm, and a decrease at 488 nm; therefore, the zinc-dependent probe excitation properties are very similar to those occurring after the direct addition of H_2_O_2_ (Figure 3b). 

We also added micromolar zinc to growing cultures of *S. cerevisiae* and *S. pombe,* where toxicity was not observed. We detected a small but reproducible oxidation of the biosensor, which was very transient and maximal at all concentrations tested, ranging from 1 to 100 µM zinc (Figure 3c,e). Again, the excitation properties of the biosensor upon zinc administration were very similar to those upon peroxide addition, suggesting that intracellular H_2_O_2_ had been generated (Figure 3d,f). 

### 3.4. In Wild-Type Fission Yeast, Non-Toxic Zinc Causes a Sudden H_2_O_2_ Burst by Enhancing Cu/Zn Superoxide Dismutase Activity

**Figure 4:**
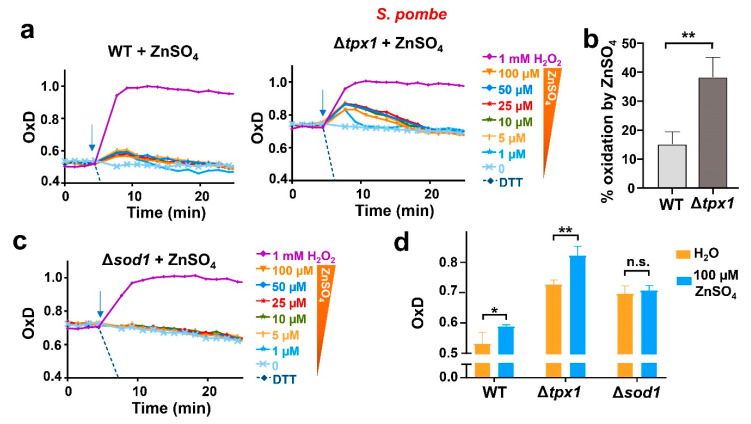
In fission yeast, non-toxic levels of zinc cause a sudden H_2_O_2_ burst which depends on the presence of Sod1. (**a**,**c**) Cultures from strains (**a**) HM123 (WT), SG5 (Δ*tpx1*), and (**c**) JM18 (Δ*sod1*), transformed with p407.C169S to express roGFP2-Tpx1.C169S, were grown, treated, and processed as described in Figure 3. Average data from three biological replicates are shown, and error bars (S.D.) are displayed in Figure S3. In all graphs, the time of addition is indicated with arrows. (**b**) Percentage of probe oxidation upon 100 µM ZnSO_4_ in WT and Δ*tpx1* at the first time point after treatment as shown in (**a**). Y-axis: the 0–100% oxidation of the biosensor in each background was determined using the OxD_0_ of each strain as 0% and 1 mM H_2_O_2_ of each strain as 100%. (**d**) The bar graph represents the OxD values of the strains in (**a**,**c**), at time 0 (orange bars) and at the first time point after treatment with 100 µM ZnSO_4_ (blue bars). Statistical significance was calculated with a ratio paired Student’s *t*-test between indicated samples with *p*-values of 0.05 (*) and 0.01 (**); n.s., non-significant.

We decided to continue studying the generation of H_2_O_2_ from added zinc using fission yeast, for which we have a vast collection of strains of known impact on peroxide homeostasis. Thus, we applied various concentrations of the metal to cells lacking the main H_2_O_2_ scavenger Tpx1. We had shown before that roGFP2-Tpx1.C169S expressed in Δ*tpx1* is able to sense concentrations of peroxides lower than in a wild-type background since H_2_O_2_ is not scavenged, and the basal level of oxidation of the probe, OxD_0_, is also significantly altered (20% more oxidized than in wild-type cells) [27]. As shown in Figure 4a,b and Figure S3, the oxidation of the sensor upon the addition of micromolar zinc was quantitatively larger than that observed in a wild-type background, confirming that H_2_O_2_ had been intracellularly generated as a secondary event upon zinc supplementation.

The fact that all the concentrations of zinc used were capable of triggering a non-proportional and similar burst of H_2_O_2_ production suggested that the metal had been able to activate peroxide production from a limited cellular source. This could be the case of the cytosolic copper/zinc superoxide dismutase 1, Sod1. This enzyme is essential to dismutating superoxide anion into H_2_O_2_ and oxygen, and it is located in the cytosol and the intermembrane mitochondrial space [40,41]. Mutations in the gene coding for Sod1 are linked to amyotrophic lateral sclerosis (ALS), probably through the effect of mutations on the cellular proteostatic balance [42,43]. The concentration of Sod1 in *S. pombe,* based on quantitative analysis of the fission yeast proteome, is very high under basal conditions, in the order of 4–5 µM [44]. As mentioned above, the standard minimal media used for the growth of *S. pombe* contains 1.4 µM zinc, and even though there are plasma membrane transporters importing the metal against the gradient, it is reasonable to believe that cells could have a small fraction of the abundant Sod1 without zinc and that this pool would transiently benefit from an extra supply of zinc added to the media. To test whether Sod1 is the source of the transient H_2_O_2_ production, we expressed the biosensor roGFP2-Tpx1.C169S in cells lacking Sod1. As shown in Figure 4c,d, zinc addition to the media did not cause probe oxidation in Δ*sod1*, indicating that Sod1 was the source of H_2_O_2_ in wild-type cells upon the addition of extra zinc. 

## 4. Discussion

The use of reliable methods to measure fluctuations of H_2_O_2_ in cells in culture may help decipher fundamental questions in redox biology. Here, we expressed the peroxide biosensor roGFP2-Tpx1.C169S in three model systems (fission and budding yeasts and mammalian Jurkat cells). By doing so, we demonstrate that some general principles regarding H_2_O_2_ homeostasis govern the three models. 

We have used a fluorescence plate reader to measure changes in the fluorescence properties of the biosensors from cell suspensions. To avoid quenching, we had to optimize the growth media of fission yeast (i.e., sterilizing by filtering instead of autoclave) [27,45]. We have not been able to find growth media for Jurkat or *S. cerevisiae* compatible with the *in-culture* readings described here. Instead, cell suspensions of these two cell types compatible with fluorescence recording were accomplished by resuspending cells in isotonic solution (for Jurkat) or in *S. pombe* filtered MM (for budding yeast). Even though the viability of these cell suspensions is maintained during the short time lapses (20–30 min in most experiments), we cannot dismiss that some of the observations reported here are caused by the lack of growth under these conditions. Thus, the quantitatively high H_2_O_2_ production caused by zinc added to Jurkat cell suspensions could be a consequence of the not fully physiological solution. Nevertheless, it is worth mentioning that oxidation of the biosensor HyPer has been reported in human airway epithelial cells using the addition of zinc plus an ionophore, which exacerbates zinc availability [46]. In our case, 100 µM zinc, causing a significant increment in intracellular H_2_O_2_ concentrations according to our biosensor (Figure 3), has been described to exert toxicity to Jurkat cells [8]. Along the same lines, oxidation of the biosensor upon extracellular peroxides in *S. cerevisiae* seems to be slightly different from the other two models, suggesting an apparent steeper gradient of extracellular-to-intracellular peroxides in budding yeast relative to *S. pombe* and Jurkat; again, probe oxidation in *S. cerevisiae* was performed using sub-optimal media. Further experiments will have to be performed to confirm or dismiss this small difference in plasma membrane permeability.

The response of Jurkat to extracellular zinc regarding H_2_O_2_ generation is dramatically different from that of the yeast models. Notably, the sensitivity to this metal is also very different among the different cell types, with zinc being toxic only at very high concentrations of the metal in the yeasts [9,16], while moderate concentrations are sufficient to exert toxicity to Jurkat [8]. The high levels of H_2_O_2_ generated by zinc in Jurkat can explain the observed toxicity or be a consequence of it. A possibility for the different effects of zinc in these model systems is that the cellular regulation of transporters (from the extracellular to the intracellular media, and from the cytosol to the vacuole/lysosome/ER) are radically different, but further experimental evidence will be required to support this hypothesis.

In both yeast models, moderate concentrations of zinc did not seem to cause a toxic wave of intracellular H_2_O_2_. Instead, a moderate and transient increase in peroxides was detected in both model systems, suggestive of the sudden activation of a dormant source of H_2_O_2_, followed by homeostatic scavenging of the produced peroxides by the antioxidant cell defense. Using fission yeast, we have shown here that a minor fraction of the abundant Sod1 may exist in a zinc apo-form when grown in minimal media. In fact, it was previously reported that the addition of extracellular zinc to fission yeast cultures causes an enhancement of catalytically active Sod1 [16]. Interestingly, in human cells it has been proposed that excess zinc may alter mitochondrial function and produce superoxide that could favor superoxide dismutase activity [21]; this is unlikely to occur in fission yeast, where the effect of zinc on H_2_O_2_ production is non-toxic, non-proportional to the dose, and transient. In conclusion, moderate supplementation of zinc to cell suspensions may be generally beneficial for maximizing Sod1 cellular activity and for bursting the antioxidant cell capacity.

## 5. Conclusions

Thanks to the use of the reporter roGFP2-Tpx1.C160S expressed in *S. pombe*, *S. cerevisiae*, and Jurkat cells, we have here generalized a previous findings described previously in fission yeast: (i) the peroxide gradient from extracellular media to the cytosol is around 300:1, and (ii) the steady-state levels of peroxides are in the low nanomolar range. Furthermore, we have also demonstrated that the addition of the redox-inactive metal zinc can cause fluctuations in intracellular peroxides, which are transient and limited in both yeasts, and which are probably caused by activation of an apo-Sod1 pool lacking intra-molecular zinc. The future expression of similar biosensors in other sub-cellular compartments (mitochondrial matrix, intermembrane space, or endoplasmic reticulum) in the three model systems will be required to continue generalizing basic redox biology principles related to peroxide fluctuations.

## Data Availability

The data presented in this study are available in the article and Supplementary Materials.

## Supplementary Materials

The following supporting information can be downloaded at: https://www.mdpi.com/article/10.3390/antiox12030706/s1, 3 supplementary figures S1 to S3. Figure S1: Expression of roGFP2-Tpx1.C169S in fission yeast, Jurkat and budding yeast; Figure S2: Cell suspensions of HM123 (*S. pombe*), Jurkat T cells or BY4741 (*S. cerevisiae*) (**c**) expressing roGFP2-Tpx1.C169S; Figure S3: Fission yeast strains HM123 (WT), SG5 (Δ*tpx1*) and JM18 (Δ*sod1*), transformed with p407.C169S.
