# Supplementary material for: Expression of the H2O2 Biosensor roGFP-Tpx1.C160S in Fission and Budding Yeasts and Jurkat Cells to Compare Intracellular H2O2 Levels, Transmembrane Gradients, and Response to Metals
Source: Antioxidants (Basel). 2023 Mar 13;12(3):706. doi: 10.3390/antiox12030706 (PMC10045392; doi:10.3390/antiox12030706)
Supplement: Supplementary file 1 [file antioxidants-12-00706-s001.zip › antioxidants-2236835-supplementary.pdf]

SUPPLEMENTARY DATA

Expression of the H_2O_2 biosensor roGFP-Tpx1.C160S in fission and budding yeasts and Jurkat cells to compare intracellular H_2O_2 levels, transmembrane gradients and response to metals

de Cubas et al.

It includes:

3 supplementary Figures

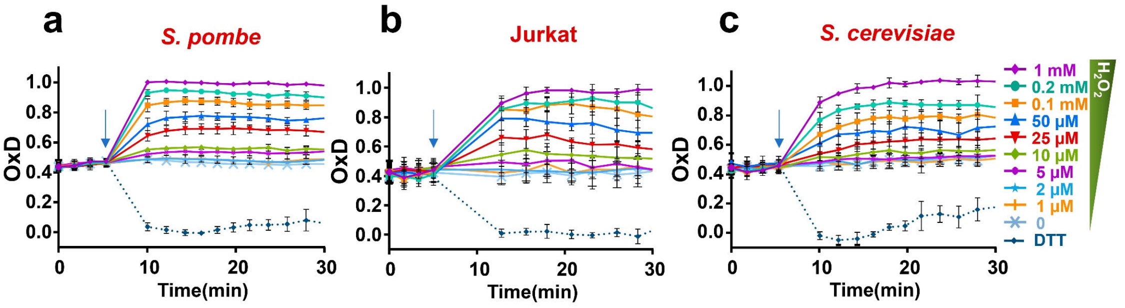

Figure S1. Expression of roGFP2-Tpx1.C169S in fission yeast, Jurkat and budding yeast. Wild-type of *S. pombe* (HM123), *S. cerevisiae* (BY4741) or Jurkat T cells, were transformed with plasmids p407.C169S, p791 or stably expressed p797, respectively, all allowing roGFP2-Tpx1.C169S expression. Oxidation of the reporter was estimated as described in Figure 2. Data from three biological replicates with error bars (S.D.) are shown. The time of addition is indicated with arrows.

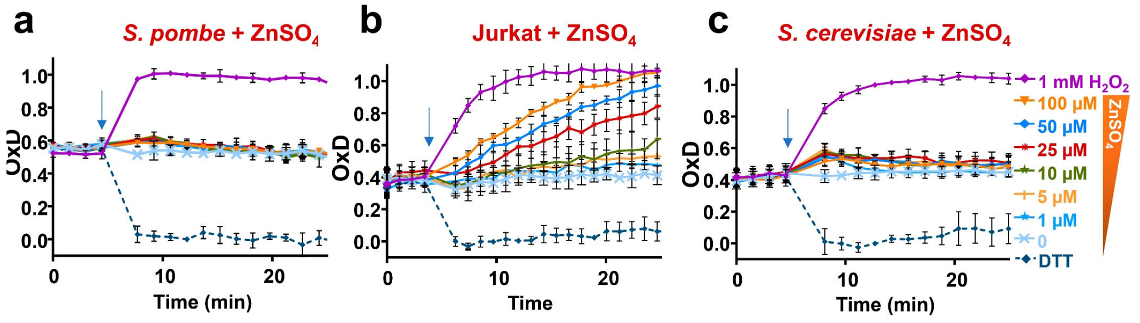

Figure S2. Cell suspensions of HM123 (*S. pombe*) (**a**), Jurkat T cells (**b**) or BY4741 (*S. cerevisiae*) (**c**) expressing roGFP2-Tpx1.C169S were treated with H_2O_2 or DTT (as controls) or with the indicated concentrations of $ZnSO_4$. Experiment and analysis was performed as described in Figure 3. Data from three biological replicates with error bars (S.D.) are shown. The time of addition is indicated with arrows.

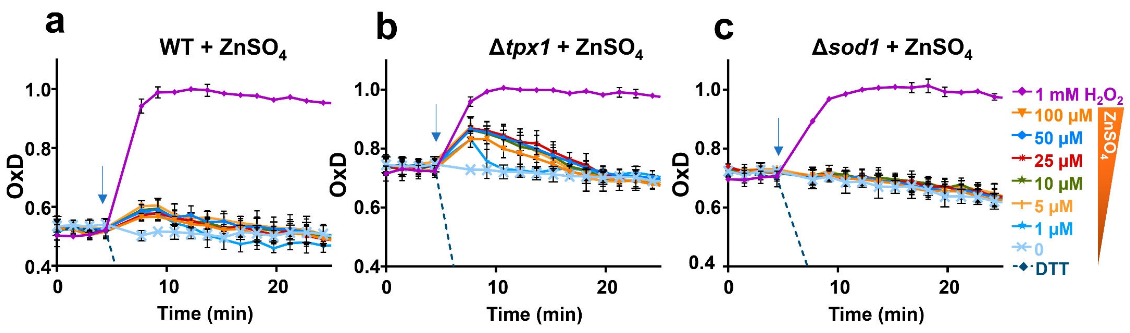

Figure S3. Fission yeast strains HM123 (WT) (**a**), SG5 (Δ*tpx1*) (**b**) and JM18 (Δ*sod1*) (**c**), transformed with p407.C169S to express roGFP2-Tpx1.C169S were treated with the indicated concentrations of DTT, $ZnSO_4$ or H_2O_2. Experiment and analysis was performed as described in Figure 4. Data from three biological replicates with error bars (S.D.) are shown. The time of addition is indicated with arrows.
